# The Recent Trend in the Use of Multistrain Probiotics in Livestock Production: An Overview

**DOI:** 10.3390/ani11102805

**Published:** 2021-09-26

**Authors:** Modinat Tolani Lambo, Xiaofeng Chang, Dasen Liu

**Affiliations:** 1 College of Animal Science and Technology, Northeast Agricultural University, Harbin 150030, China; motolanilambo@neau.edu.cn (M.T.L.); changxiaofeng629@163.com (X.C.); 2 College of Science, Northeast Agricultural University, Harbin 150030, China

**Keywords:** gut microbes, feed additives, growth performance, cattle, chicken, pigs

## Abstract

**Simple Summary:**

Probiotics are live microorganisms that confer a health benefit to the administered animal when ingested. Their use has been an effective alternative to antimicrobial growth promoters in the livestock industry, and they could be bacteria or non-bacterial species or strains. However, there has been an increasing trend in administering multistrain probiotics in recent years. Multistrain probiotics comprise two or more species or strains of important microorganisms as a consortium beneficial to the administered animal. Several studies are being carried out to explore their potency or efficiency. They have proven to be a promising alternative to antibiotics growth promoters and were responsible for enhancing gut health, growth performance, maintaining a balance in gut microbiota, stimulating immunity against pathogenic organisms, improving digestion, and overall production efficiency in ruminants, poultry, and swine production.

**Abstract:**

It has been established that introducing feed additives to livestock, either nutritional or non-nutritional, is beneficial in manipulating the microbial ecosystem to maintain a balance in the gut microbes and thereby improving nutrient utilization, productivity, and health status of animals. Probiotic use has gained popularity in the livestock industry, especially since antimicrobial growth promoter’s use has been restricted due to the challenge of antibiotic resistance in both animals and consumers of animal products. Their usage has been linked to intestinal microbial balance and improved performance in administered animals. Even though monostrain probiotics could be beneficial, multistrain probiotics containing two or more species or strains have gained considerable attention. Combining different strains has presumably achieved several health benefits over single strains due to individual isolates’ addition and positive synergistic adhesion effects on animal health and performance. However, there has been inconsistency in the effects of the probiotic complexes in literature. This review discusses multistrain probiotics, summarizes selected literature on their effects on ruminants, poultry, and swine productivity and the various modes by which they function.

## 1. Introduction

With the rapidly increasing demand for animal food products globally, improving livestock productivity to meet the growing demand has become important to livestock producers [1]. Feed additives provide a safe and healthy way to enhance animal feed and improve livestock productivity, health, and general well-being. Due to the development and spread of antimicrobials-resistant bacteria, which may threaten the health of animals and consumers of animal products, antibiotic growth promoters have been questioned for use as a livestock feed additive. The European Union has, in “Regulation (EC) No 1831/2003 of the European Parliament and of the Council of 22 September 2003 on additives for use in animal nutrition”, prohibited the use of antimicrobial drugs and ionophores as growth promoters in animal production since 2006 [2]. As a result, there was a need for alternate therapeutic and prophylactic options. The research spotlight has been on probiotics, prebiotics, symbiotics, and immunomodulators as antibiotics alternatives in the animal husbandry industry to improve livestock health and maintenance; nonetheless, probiotics have remarkably met the expectations of livestock breeders [3].

Despite the fact that probiotics are often used interchangeably with direct fed microbials (DFM), there is a slight difference in their definition regarding animal feeding [4]. The U.S. Food and Drug Administration has defined DFMs as feed products that are believed to contain or are a natural source of viable microorganisms [4,5,6]. On the other hand, various definitions have been used to describe probiotics. They were initially defined as substances produced by a protozoan and then stimulated by another; they were then defined as feed additives that exert advantageous effects by modulating the intestinal microbial ecology of the administered host [7]. In 2002, the Food and Agriculture Organization (FAO) defined them as “live microorganisms that provide health advantages to the host when administered in appropriate doses” [8], while the International Scientific Association in 2013 updated the definition as “live microbials of strictly selected microorganisms which, when administered in adequate amount confer a health benefit to the host” [9,10,11]. They are described as non-toxic, non-pathogenic, and generally recognized as safe. In the past few years, they have been acknowledged as supplements or feed additives and antibiotic alternatives in the livestock industry based on the speculation that ingesting high levels of certain beneficial bacteria could inhibit the growth of pathogenic bacteria and prevent the digestive tract from pathogenic invasion [12]; this is coupled with the fact that they do not deposit dangerous residual substances or pose adverse side effects on the administered host [3]. Their passage through the gastrointestinal tract of animals influences the intestinal microbiome quantitatively and qualitatively, modifying the immune system and improving health and productivity.

**Figure 1:**
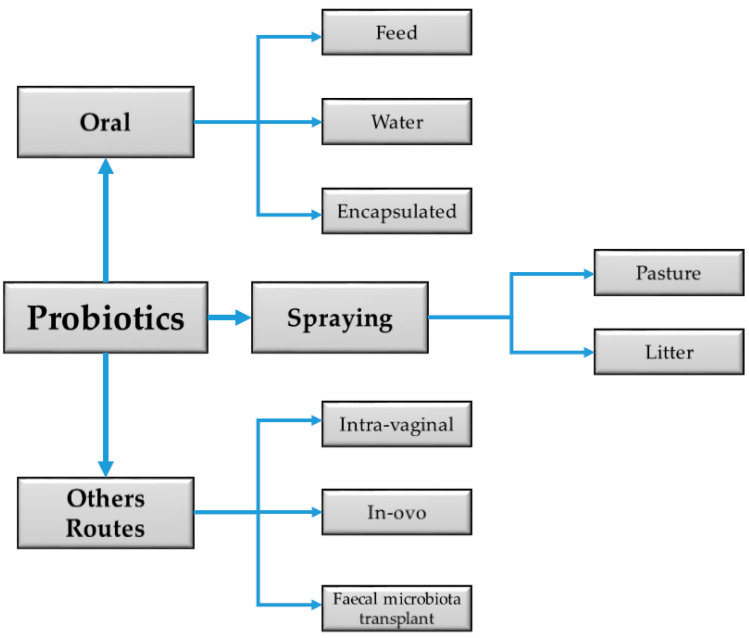
Diagram summarizing the common routes of administering probiotics in livestock.

Probiotics preparations come in various forms, and their efficacy sometimes varies depending on whether they are mono- or multistrain. The new approach in probiotics utilization has been to use a combination of probiotics strains. This strategy is presumed to have highly influenced animal nutrition, exerted increased health benefits, and created an even more favorable balance of intestinal metabolism, animal welfare [13], and performance than single-strain cultures [14]. They can be administered via several routes (Figure 1), but the oral method is most common in animal husbandry.

## 2. Common Probiotic Strains and Their Mode of Action

Bacteria, bacteriophages, microalgae, and yeasts are all examples of probiotics [15]. Although numerous microorganisms have probiotic potential, *Lactobacillus*, *Streptococcus*, *Enterococcus*, *Lactococcus*, and *Bifidobacteria* remain the most commonly used probiotic agents in livestock to date [14,15,16]. *Saccharomyces* (*S. cerevisiae* and *S. bourlardii*), *Candida pintolopesii*, and *Aspergillus oryzae* are typical non-bacterial probiotics [16,17,18]. There are currently numerous commercially available mono- and multistrain probiotics [19]. Some authors have included inactivated microorganisms, describing them as “live or dead bacteria, or components of bacteria (such as cell walls) that work under multiple modes of action, conferring positive effects to the administered animal or its environment” [20]. Before registering a strain as probiotic, specific criteria must be ensured and documented, such as its ability to survive and be preserved in the digestive tracts during the passage [21], non-pathogenicity and toxicity, lack of undesirable side effects, stability, large-scale production potential, and beneficial clinical effects on the administered animals [22,23,24]. Potential candidates should be able to modify specific physiological parameters or the immune system, attenuate pathogens, treat and prevent infections, inflammation, and disease while also acting as a biological control to prevent spoilage [22,25]. Hill et al. [26] noted that they must contain specified contents, appropriate viable count at the end of shelf life, and established evidence for health benefits. Most importantly, they must be “safe for their intended usage.” According to current bacterial nomenclature, the “International Code of Nomenclature” should be used in naming or classifying new probiotic strains [27].

Even though probiotics are considered a possible replacement for antibiotic growth promoters, their mode of action appears to be distinct [28]. Probiotics impacts are species-specific [29] and may also rely on the physiological and immunological condition of the administered animal. Different probiotics exert their benefits via mechanisms yet to be fully understood but are presumed to be related to their gastrointestinal lumen or wall activities. Their primary function results from the production of a range of antibacterial and bacteriostatic substances, such as organic acids, bacteriocins, diacetyl, antibiotics, and hydrogen peroxide [3], which exert beneficial effects through three primary pathways [30]:

(1) Competitive exclusion,
(2) Bacterial antagonism, and
(3) Immune system stimulation.

Probiotics also impact the health of the administered host via competition between beneficial bacteria and pathogens, replacement of pathogens by probiotic bacteria, and regulation of innate and adaptive immunity [31]. Due to their antagonistic effect, probiotics can hinder the growth of noxious bacteria by altering the gut microbiome, reduce the spread of pathogens and their emission during infection, decrease gut permeability, ameliorate clinical symptoms in livestock, boost immunity, and improve disease resistance and health [32,33,34]. In addition, they appear to be effective in foodborne pathogen reduction, for example, *Salmonella*, *Escherichia coli*, *Campylobacter*, *Clostridium*, *Staphylococcus aureus*, and *perfringens* [35,36], hence improving intestinal digestion and nutrient absorption and supporting a healthy micro ecological state. They can even aid pollution reduction by preventing the accumulation of harmful chemicals and lowering ammonia emissions in animal manure [37,38].

## 3. Advent of Combining Microorganisms

The idea of using harmless bacteria to out-compete pathogens has been acknowledged for a long time [39]. In 1907, a Russian scientist, Ellie Metchnikoff, suggested that ingested bacteria could positively influence the normal microbial flora of the intestinal tract [40]. Later in 1908, he coined the term probiotic from two Greek words, “pro” and “bios”, meaning “for life.” Probiotic utilization has continued to grow over the years, and single probiotic strains are assumed to have multiple effects on their host [39]. However, in 1992, a panel of experts stated that mixed microbial cultures are optimal as prophylactics [41]. Famularo et al. [42] hypothesized that the chances of effectively colonizing the gastrointestinal tract by a single strain microorganism might be lower. Dunne et al. and Rolfe [43,44], in 1999 and 2000 respectively, proposed that probiotics could consist of two or more microorganism combinations. The idea is to combine two or more strains of the same species, genus, or several genera of bacteria, sometimes including some fungal species like *Saccharomyces* [45] that could play distinct functions in the microbial processes, since the different strains could have different target at the delivery site and complement each other’s effect on their host [39].

Famularo et al. [42] probed the likelihood of genetic exchange between probiotics and the gut microflora. The genetics of the species or strains of multistrain probiotics are key in understanding the principle by which they interact with each other, the intestinal microbiota, and the administered host. The mechanism whereby they exert more advantages is mostly connected to synergism, antagonism, and additive effect of the multiple strains, which culminates in high adherence to the gut mucosae and hinders the colonization of pathogens. Douillard et al. [46] proved that genes coding various bioactive compounds such as bacteriocins, antibacterial peptides, lectins, and bioactive proteins are present in the probiotics genome. Bacteriocins, as an example, are produced by Gram-positive and -negative bacteria [47], and their efficacy has been established in inhibiting pathogenic bacteria [48,49]. They could also be antagonistic towards closely related strains. As a result, these compounds are connected to the antagonistic function of complex probiotics in inhibiting pathogenic bacteria or fungi present in the gastrointestinal tract. In addition, the abundance of fimbriae, which are thin protein structures located on some bacteria’s cell surface, enables them to bind to the gut epithelium [45], enhancing the interaction of the isolates with each other and the host cells.

These microbial consortia can thrive in a constantly changing environment such as the gastrointestinal tract and regulate the resident microbiota. Due to this fact, a multistrain probiotics supplement has been advocated as being more effective than a monospecies supplement [50,51,52].

For instance, even while some monostrain probiotics are effective in treating digestive tract disorders, Sanders et al. [53] noted that multistrain probiotics might be more effective in amplifying the protective spectrum against microbial infections. It has been shown by previous in vitro studies that the combined effects of several strains could manifest superior inhibitory effects on enteric pathogens [54].

Meanwhile, the potential of their cell walls to absorb heavy metals [55] enables some multistrain probiotics to reduce the absorption of harmful chemicals in animals [56]. This has prompted their use in dietary supplements, detoxification therapy, and biotechnology [56,57]. They have shown significant efficacy in stimulating the immune system and function [58], competing against other microorganisms for nutrients, performing bactericidal and antibacterial activity, and competing on the adherence site for space [54]. Their performance is, perhaps, more consistent and efficient [39,59]. Examples of commercial multistrain probiotics include, PoultryStar ME, containing *Pediococcus acidilactici*, *Lactobacillus reuteri*, *L. salivarius*, and *Enterococcus faecium* [60]; PrimaLac containing *Bifidobacterium thermophilum*, *E. faecium*, and *Lactobacillus* spp [18]; and Microguard containing various species of *Bacillus*, *Lactobacillus*, *Saccharomyces*, *Bifidobacterium*, and *Streptococcus* [61]. In general, it appears that the *Lactobacillus* groups are significant constituents of a probiotic mix.

## 4. Multistrain Probiotic Use in Ruminants

The ruminants’ gastrointestinal tract is estimated to inhabit over 5000 microorganism species [62], with the rumen, described as the ruminants’ “Black Box” [63], having the most diversified population of anaerobic bacteria, fungi, archaea, protozoa, and viruses [64]. Various health challenges could stem from an unhealthy or imbalanced gut microbiome. Several novel approaches in improving the microbiome of ruminants’ digestive tracts, particularly the rumen, are being investigated. Several studies have shown that probiotics can help increase milk quality, improve growth performance, increase average daily weight gain, improve feed efficiency, and reduce diarrhea in ruminants [63,65,66,67,68,69].

At the onset of diarrhea in dairy calves, a multispecies probiotic containing five bacteria strains (*Bifidobacterium bifidum*, *Pediococcus acidilactici*, *Lactobacillus acidophilus*, *Lactobacillus casei*, *Enterococcus faecium*), peptide extract, dead yeast extract, dried whey, an enzyme blend, and natural flavor rapidly resolved the condition by reducing the duration of symptoms. The calves’ daily weight gain improved with the combination as well [70]. Buffaloes supplemented with a multistrain probiotic-containing six bacterial strains (*Streptococcus faecium*, *Lactobacillus casei*, *Lactobacillus acidophilus*, *Lactobacillus bulgaricus*, *Lactobacillus reuteri*, *Lactobacillus lactis*) and two yeast strains (*Aspergillus oryzae*, *Saccharomyces cerevisiae*) had no improvement with respect to body condition score and dry matter intake but had a higher average daily milk yield, and reduced feed conversion ratio [71].

Furthermore, Kembabazi et al. [72] discovered that a mixture of *Lactobacilli plantarum* and *Saccharomyces cerevisiae* could operate as a probiotic. According to the findings, the mechanism by which they exert their probiotic function involves producing a low and stable lactate concentration in the rumen, resulting in a low pH medium suitable for the activity of *S. cerevisiae*, which usually amplifies the rumen bacteria population and competes against starch-utilizing bacteria. Owing to the potentiality of yeast to regulate pH and scavenge oxygen, they limit lactate build-up, creating a more conducive habitat for the cellulolytic activity of bacteria. Therefore, resulting in enhanced fodder consumption [73] as indicated by improved dry matter intake in nursing dairy cows.

In another study, Olchowy et al. [74] top-dressed pasture with a liquid commercial probiotic product containing a mixture of multispecies constituting four bacteria strains (*Lactobacillus rapi*, *Lactobacillus parafarraginis*, *Lactobacillus zeae*, and *Lactobacillus buchneri* with a minimum concentration of each strain, 10^6^ CFU/mL), *Acetobacter fabarum* (minimum concentration of 10^5^ CFU/mL) and yeast from the environment (*Candida ethanolica*; minimum concentration of 10^6^ CFU/mL). Based on the result, cows that grazed pasture treated with the product produced a significantly higher volume of milk and a higher quantity of milk protein with tendencies towards producing more milk fat. Similarly, when dairy cows were directly fed the pasture from paddocks treated with the same probiotic mixture, the treatment group still produced more milk and higher milk protein content than the control group. In addition, Deng et al. [75] used an intravaginal infusion to give transition dairy cows a lactic acid bacteria cocktail containing *Lactobacillus sakei*, *P. acidilactici* FUA3138, and *P. acidilactici* FUA3140 combinations around parturition. The result revealed lower non-esterified fatty acids, higher cholesterol, and higher lactate levels, indicating that the concentrations of specified metabolites in the blood serum of transition dairy cows had been altered. A summary of several other combinations used in cattle, sheep, and goat of different physiological status and age are presented in Table 1.

## 5. Multistrain Probiotic Use in Poultry

Pathogenic bacteria including *E. coli*, *Clostridium*, and *Salmonella* appear to be a severe concern in chicken production, causing mortality, lowered growth rate, and low output. Antibiotics had previously played an important role in combating or regulating this problem; however, their prohibition has resulted in the use of probiotics to fill the void. Generally, because of their high fermentation utilization activity, probiotics promote protein and lipid digestion and interacts with enzymes to break down dietary molecules into simpler forms for digestion and absorption. They stimulate the production of digestive enzymes for carbohydrate metabolism, lower cholesterol, help in the synthesis of nutrients such as vitamins, influence the pH level in the poultry gut, and improve the productive performance, intestinal flora, and histomorphometry in heat-stressed chickens [37,85,86,87].

When broiler chickens were experimentally challenged with *Pasteurella multocida*, a highly contagious poultry disease that causes fowl cholera [88,89], supplementing dietary multistrain containing *Saccharomyces cerevisiae*, *Lactobacillus fermentum*, *Pediococcus acidilactici*, *Lactobacillus plantarum*, and *Enterococcus faecium* improved feed efficiency, growth performance, and intestinal health. It mitigated clinical signs, inflammatory reactions, and mortality-related symptoms [62]. In previous studies, successes have been recorded on probiotics’ potency in attenuating the colonization of avian pathogens in the chicken gut [60,90,91,92,93]. These antimicrobial effects are traceable to bacteriocins, organic acids, hydrogen peroxide, and short-chain fatty acids secreted by probiotic bacteria [94]. Besides, the transcriptional profiles of anti-inflammatory genes in the intestinal mucosa of probiotic-fed birds were elevated, haemato-biochemical markers such as packed cell volume, total cholesterol, glucose, proteins, white blood cells, and lymphocytes were also improved. There is a possibility that perhaps the synergy between lactic acid bacteria and yeast strains resulted in higher antimicrobial activity against *P. multocida* and *enterobacteria* in the guts of supplemented birds, as well as the ability of the combination to out-compete pathogens, thereby preventing them from attaching to the intestinal walls and as a result improve intestinal microbial balance [95].

Furthermore, Kazemi et al. [96] fed two commercial multistrain probiotic products to broiler chicks in another investigation. The first contains seven bacteria strains (*Enterococcus faecium*, *Lactobacillus d. bulgaricus*, *Lactobacillus acidophilus*, *Lactobacillus plantarum*, *Lactobacillus rhamnosus*, *Streptococcus s. thermophiles, Bifidobacterium bifidum*) and two fungi (*Aspergillus Oryza* and *Candida pintolopesii*), while the other contains nine bacteria strains (*Enterococcus faecium*, *Bifidobacterium bifidum*, *Lactobacillus casei*, *Lactobacillus rhamnosus*, *Lactobacillus plantarum*, *Pediococcus acidilactici*, *Bacillus subtilis*, *Lactobacillus acidophilus*, and yeast (*Saccharomyces cerevisiae*). Both products improved broiler chicken overall performance, enhanced intestinal structure, reduced lipid peroxidation, increased the population of *lactobacillus* in the ileum, and lowered *clostridium* spp. The probiotics strains could have lessened the impairment of the intestinal epithelium [72] and reduce the abundance of *clostridium* by competing for nutrients at the adherence site and inducing the immune response.

In spite of the numerous benefits associated with the administration of multistrain probiotics, not all have shown significant benefits [54] (Table 2). For example, there have been reports of no effect on broiler breeder performance, gastrointestinal tract function, cholesterol concentrations, cell-mediated immunity, malondialdehyde, serum glutathione peroxidase activity, and blood hematology with certain probiotic mixtures containing a dose of 2.5 × 10^7^ CFU/g *Bifidobacterium thermophilum*, *Lactobacillus casei*, *Lactobacillus acidophilus*, and *Enterococcus faecium* [83,97]. Nevertheless, their effectiveness is yet preferred compared to their single strain counterpart [58]. The role played by dosage in their efficacy cannot be overemphasized. Dobrowolski et al. [98] investigated the optimal dose of probiotics preparation containing four mixed bacterial strains and yeast isolate to improve turkey poults’ small intestine development and structure. In this study, three doses of 10^7^ CFU/g, 10^8^ CFU/g, and 10^9^ CFU/g in an amount of 500 g/1000 kg were administered to different groups of birds. Although all the doses were said to benefit the intestinal structure, the intermediate dose accelerated the development of the GIT, especially the duodenum. It would be expected that a higher dose would exert more benefits, but this was not the case because the highest dose had a poorer outcome. Hence, animal dose–response to probiotics, especially the probiotic complexes, remains a critical issue to be addressed.

## 6. Multistrain Probiotic Use in Swine

Feed prices contribute to almost two-thirds of overall swine production expenses; hence, to ensure profitability in the pig industry, efficiency in converting feed into pig body mass is essential [108]. Moreover, improved metabolic utilization of dietary nutrients is dependent primarily on a healthy gut, which can lead to improved feed digestion and nutrient absorption [109]. Research has shown that multistrain probiotics could enhance growth performance, feed efficiency, and nutrient digestibility [110,111,112]. It has also been effective in maintaining a balance in the intestinal microbial flora [113,114], stimulating immunity [76,115], increasing litter size, vitality, and weight, and reducing fecal noxious gas emission in pigs [111,112]. A summary of the effects of some multistrain probiotics on pigs of different physiological statuses is presented in Table 3.

In piglets, a study using a blend of bacteria probiotics containing *Lactobacillus salivarius*, *Lactobacillus reuteri* (VB4), *Lactobacillus reuteri* (ZJ625), and *Streptococcus salivarius* as direct-fed microbial showed that the combination had a positive impact on growth performance and blood profile. The combination elevated average daily weight gain, reduced feed conversion ratio, reduced the population of ileal enteric bacteria, and activated immunoglobulin G in weaned piglets, indicating its efficacy in preventing post-weaning diarrheal disorders [77]. Further, Lan et al. [97] discovered that supplementing pigs’ diets with multistrain probiotics composed of spray-dried spores of *Clostridium butyricum*, *Bacillus lichenformis*, *Bacillus coagulans*, and *Bacillus subtilis* increased average daily weight gain and feed ratio, and also reduced hydrogen sulfide and total mercaptans emission (a fecal noxious gas of environmental concern). A higher dose at 0.1% increased apparent total tract digestibility of dry matter, nitrogen, and gross energy. It also modulated the fecal *lactobacillus* count and reduced the *E. coli* population. Similarly, with multistrain containing 1 × 10^9^ CFU/g *Bacillus subtilis* and *Bacillus licheniformis*, Hu et al. [116] reported an increased average daily weight gain and total body weight of piglets, increased apparent total tract digestibility of dry matter, and reduced mercaptans, ammonia, and *E. coli* in the lactating sow.

Besides, the performance of breeding sows during the reproductive phase is often influenced by stressors such as gestation, farrowing, lactation, and weaning [117]. Hayawaka et al. [116] proved that multistrain probiotics comprising *Enterococcus faecalis*, *Clostridium butyricum*, and *Bacillus mesentericus* administered 3 weeks before farrowing improved the rate of return of sows to oestrus by 24% and reproduction performance during the farrowing periods. Bohmer et al. and Alexopoulos et al. [118,119] suggest that the immune system’s stimulation or modulation of the gut microbiota is the possible mechanism for the outcome. In spite of this, Arsene et al. [35] reported no effect of *Bacillus licheniformis* and *subtilis* combination on the reproduction performance of lactating sows. Such variations in the effectiveness of probiotics, probably due to the complexity of the livestock digestive system or differences in the strains or species combined, remain unraveled.

## 7. Conclusions

In ruminants, poultry, and swine, multistrain probiotics have proven to be a viable alternative to antibiotics, and their usage in animal husbandry continues to grow. The effect on and responses of host animals, however, differs among literature. The variability in results might be due to the microorganism type or strains combined, as different species could possess distinct metabolic effects. The survivability of all the strains until delivery to the gut may also be difficult to ascertain. Probiotic dosage, the number of viable organisms in each dose, host animal physiological status and age, environment, diet composition, production procedures, and the mode of administering to the animal could all have a role. There were also limited reports on the greater benefits of multistrain probiotics over single strains in livestock. As a result, further research is needed to understand the interaction mechanisms among the combined microbes and the host’s gut microbiota and the unique role played by the individual microbe. In addition, comparison among the investigated animals and direct comparisons between the mono- and multispecies probiotics should be considered. Finally, stringent recommendations for optimal benefits should be provided.

## Figures and Tables

**Table 1:** Various combinations of multistrain probiotics and their effect on ruminant production.

Multistrain	Cell Count	Mode of Administration/Dose	Host	Duration	Effect	No Effect	Ref.
*Bacillus foraminis*, *B*. *firmus* *B*. *licheniformis*, *Staphylococcus saprophyticus* *bovis*	10^7^ CFU/g	Oral inoculant using a syringe (1 mL/day at 1–2 weeks, 2 mL/day at 3–9 weeks)	Neonate lamb	9 weeks	Reduced feed intake Lower acetate to propionate ratio	No effect on BWG and wool quality	[76]
*P. acidilactici 3G3* *L. plantarum BS* *S. cerevisiae 2030*	5 × 10^9^ CFU/mL	Orally using a syringe (6 mL)	Dairy goats	9 weeks	Improved BWG, total milk fat yield, solid-non-fat, and lactose, PUN and triglyceride concentration, economic profit, and reduced somatic cell count	No effect on total milk yield, glucose, hemoglobin, and RBC count	[77]
*E. faecalis* *L. rhamnous*	2 × 10^9^ CFU/mL	Orally using dosing gun (5 mL)	South African goats	30 days	Improved weight gain Lowered gut pH Maintain the ecology ruminal microbiota	No effect on feed intake	[78]
*L. acidophilus* *L. casei* *B. thermophilum* *E. faecium*	10^7^ CFU/g	(Orally) mixed with concentrate	Lactating Ewes	8 weeks	Increased milk fat, butyric, and caproic acid	Rumen conversion pathway of Fatty acid was not altered	[79]
*L. acidophilus* *L. plantarum* *B. bifidum,* *B. subtilis,* *A. oryzae*	1 × 10^8^ 9.8 × 10^7^ 2 × 10^6^ CFU/g	Orally (3 g or 20 g/cow/day mixed with diet)	Pre-partum dairy cow	6 months	Increased DMI, milk yield and composition, serum albumin, and reduced globulin during postpartum	No effect on BW, birth weight of calves, blood biochemical concentrations	[80]
*(Locally produced probiotic bacteria) containing:* *L. farraginis* *L. reuteri* *L. rhamnosus*	10^8^ CFU/g DM	Orally (mixed with diet)	Pre-partum dairy cows	3 months	Increased feed: milk ratio, DMI, milk yield, % milk fat, and protein Enhanced postpartum uterine and cervical involution, and conception rate	No effect on milk lactose, solid non-fat, and ash	[81]
*L. casei Zhang* *L. plantarum P-8*	1.3 × 10^9^ (50 g/head/day)	Orally (mixed with basal diet)	Lactating primiparous dairy cows	4 weeks	Improved milk production and milk IgG content, lactoferrin, lysozyme, and lactoperoxidase, An increased population of rumen fermentative and beneficial bacteria Reduced somatic cell count	No effect on milk fat, protein, and lactose	[82]
*L. acidophilus* *S. cerevisiae* *E. faecium* *A. oryza* *B. subtilis*	50 mL/day	Orally (mixed with endotoxin-free water)	Dairy cows	60 days	Increased % lymphocyte Decreased neutrophil Influence genes associated with immunity and homeostasis	No effect on BW, PCV, and total protein concentration in plasma	[83]
*L. fermentum* *L. plantarum* *M. elsdenii* *S. cerevisiae*	4.5 × 10^8^ 4.5 × 10^8^ 4.5 × 10^8^ 1.4 × 10^10^	Orally (dosing of 50 mL microbial suspension)	Fattening lamb	63 days	Improved nutrient digestibility, rumen fermentation characteristics, and nitrogen retention.	No effect on feed intake and blood metabolite	[84]

BWG, Body weight gain; PCV, packed cell volume; DMI, Dry matter intake; RBC, Red blood cell; PUN, Plasma urea nitrogen; n. s, not stated by the author.

**Table 2:** Various combinations of multistrain probiotics and their effect on poultry production.

Multistrain	Cell Count	Mode of Administration/Dose	Host	Duration	Effect	No Effect	Ref.
*L. acidophilus* *L. casei* *E. faecium* *B. thermophilum*	1 × 10^8^ CFU/g	Orally 1–2 weeks; 0.9 3–4 weeks; 0.454 5–6 weeks; 0.225 g/kg Lyophilized mixture added to the diet	Male broiler chicks	42 days	Decreased gizzard weight and abdominal fat Increased antibody production	No effect on growth, carcass parameter, and blood biochemistry	[99]
*L. acidophilus* *L. casei* *E. faecium B. bifidium*	n.s	Orally (via non-chlorinated water)	Broiler chickens	42 days	Improved BW and response of antibody to new castle disease and infectious bursal disease vaccination Reduced FCR	No effect on antibody titer	[100]
*L. salivarius* *L. reuteri* *L. crispatus* *L. johnsonii*	1 × 10^5^ 1 × 10^6^ 1 × 10^7^ CFU/egg	In-ovo (100 μL/egg injected on 18th embryonic day)	Broiler chickens	1 day	Expression of cecal tonsils cytokine gene was downregulated Enhanced antibody-mediated immune responses against a highly immunogenic T cell-dependent antigen	No effect on T-cell in the spleen	[101]
*B. subtilis* CPB 011 *B. subtilis* CPB 029 *B. subtilis* HP 1.6 *B. subtilis* D 014 *B. velezensis* CBP 020 *B. velezensis* CPB 035	1 × 10^9^ CFU/g	Orally (100 g/ton mixed with feed)	*C. perfringens challenged male broiler chicken*	35 days	Improved final BW and FCR, intestinal morphology, and reduced liver weight	n.s	[102]
*L. acidophilus* *B. subtilis DSM 17299* *C. butyricum.*	2 × 10^5^ CFU/kg	Orally (mixed with diet)	*Broiler chickens*	5 weeks	Increased BW, digestibility of ileal amino acid, and humoral immune response Reduced FCR, fecal NH_3,_ and cecal *E. coli*	No effect on IgG, lymphocyte, RBC, and WBC.	[103]
*L. acidophilus* *L. casei* *B. thermophillum* *E. faecium*	2.5 × 10^7^ CFU/g	Orally (0.1 g/kg supplemented in basal diet)	*Broiler breeder (51 weeks old)*	10 weeks	Reduced ileal *E. coli*	No effect on hatchability, egg quality, mortality, fertility, BW, GIT function, or nutrient digestibility, and *Lactobacillus* spp. population	[66]
*A. oryzae* *B. subtilis* *S. cerevisiae* *L. plantarum* *Rhodopseudomonas capsulate*	1 × 10^9^ 1 × 10^9^ 1 × 10^9^ 1 × 10^9^ 1 × 10^7^ CFU/g	Orally (0.1, 0.2 and 0.4% supplemented in basal diet)	*Laying hens (40 weeks old)*	3 weeks	Improved egg protein quality	No effect on yolk color and hen productivity	[104]
*Bacillus toyonensis* *B. bifidum*	5 × 10^8^ 6 × 10^8^ CFU/mL	Orally (0.5–1 mL/kg added to basal diet)	*Japanese quail*	42 days	Enhanced growth performance, meat quality, and carcass traits Reduced feed intake, FCR, and proliferation of pathogenic intestinal bacteria	n.s	[105]
*L. casei* *L. acidophilus Bifidobacterium*	>5 × 10^9^ CFU/g	Orally (1%; 10 mL/L of distilled drinking water)	*Broiler chickens*	42 days	Improved growth performance, carcass trait, antioxidant capacity, gut microbiota, and immunity	n.s	[106]
*L. casei* *L. lactis* *L. plantarum* *Carnobacterium* *divergens* *S. cerevisiae*	10^7^, 10^8^, 10^9^ CFU/g * (3 different doses of the mixture)	Orally (500 g/1000 kg of feed)	*Meat-type female turkey*	16 weeks	Increased femur elongation and area. Reduced bone strength. Enhanced elastic strength of tibia. Influenced bone thickness.	No effect on body weight, bone mass, and bone mineral concentration	[107]

BWG, Body weight gain; FCR, Feed conversion ratio; RBC, Red blood cell; n.s, not stated by the author. * We used asteisk * to only indicate the peculiarity of the varying dosage used in this part.

**Table 3:** Various combinations of multistrain probiotics and their effect on swine production.

Multistrain	Cell Count	Mode of Administration/Dose	Host	Duration	Effect	No Effect	Ref.
*L. acidophilus* *B. subtilis* *S. cerevisiae*	1 × 10^7^ 1 × 10^7^ 1 × 10^7^ CFU/g	Orally (0.1% and 0.2% mixed with basal diet)	Finishing pigs	10 weeks	Improves ADWG and feed: gain, nutrient digestibility, growth performance, and gut microbiota Reduced serum creatinine and noxious gas emission	No effect on meat quality parameters	[117]
*Product A:* *L. plantarum L21* *L. plantarum L80* *L. paraplantarum L103* *Product B:* *B. subtilis* *L. acidophilus* *S. cerevisiae*	1 × 10^9^ 1 × 10^9^ 1 × 10^9^ 1 × 10^12^ 1.5 × 10^7^ 1 × 10^9^ CFU/mL	Oral gavage (0.25 g/day)	Weaned pigs	28 days	Increased growth performance, fecal *lactobacillus* population Reduced fecal *E. coli* Increased	n.s	[120]
*B.coagulans* *B. licheniformis* *B. subtilis* *C. butyricum*	1 × 10^9^ 5 × 10^8^ 1 × 10^9^ 1 × 10^8^ CFU/g	Orally (0.1 or 0.2 g/kg mixed with basal diet)	Growing-finishing pigs	16 weeks	Improved BW, ADWG, feed: gain ratio, nutrient digestibility, fecal *lactobacilli*, and meat quality Reduced *E. coli* and incidence of diarrhea	No effect on average daily feed intake and meat color	[121]
*L. amylovorus* *L. reuteri LAB 26* *L. reuteri LAB 49* *L. johnsonii* *L. salivarius* *L. mucosae*	1.7 × 10^19^ CFU/mL	Orally (1 mL mixed with PBS and 13% glycerol, aliquots added to feed)	Piglets	3 weeks	Increased bacteria population in the jejunum Influenced the expression of specific intestinal mucosa cytokines	No effect on the population of *lactobacilli* and bacteria in the large intestine digesta and growth enhancement	[122]
*B. subtilitis* *B. licheniformis*	1 × 10^9^ CFU/g	Orally (0.1 and 0.2% inoculated into limestone and maltodextrin as carriers)	Lactating sow and their suckling piglets	28 days	Increased piglets birth weight and ADWG Improved nutrient digestibility in sows Reduced fecal NH_3_, total mercaptans, and *E. coli* population in sows	No effect on reproductive performance, H_2_S concentration, and fecal score in sows	[123]

BWG, Body weight gain; ADWG, Average daily weight gain; n.s, not stated by the author.

## Data Availability

Not applicable.
